# Molecular characterization of the evolution of premalignant lesions in the upper aerodigestive tract

**DOI:** 10.3389/fonc.2024.1364958

**Published:** 2024-04-19

**Authors:** Axel Lechner, Jörg Kumbrink, Christoph Walz, Andreas Jung, Philipp Baumeister, Susanne Flach

**Affiliations:** ^1^Department of Otorhinolaryngology, Head and Neck Surgery, Ludwig-Maximilians-Universität (LMU) Munich University Hospital, Munich, Germany; ^2^Department of Pathology, LMU Munich University Hospital, Munich, Germany; ^3^German Cancer Consortium (DKTK), Partner Site Munich, Munich, Germany

**Keywords:** head and neck squamous cell carcinoma (HNSCC), evolution, dysplasia, premalignancy, sequencing

## Abstract

**Introduction:**

Early relapse and development of metastatic disease are some of the primary reasons for the poor prognosis of patients with head and neck squamous cell carcinoma (HNSCC). HNSCC is a heterogeneous disease which may develop in large premalignant fields of genetically altered cells. Yet knowing which individuals will progress and develop clinically significant cancers during their lifetimes remains one of the most important challenges of reducing HNSCC morbidity and mortality. To further elucidate the molecular mechanisms, we performed a focused analysis of the genome and immune microenvironment from multiple, matched normal squamous tissue, premalignant lesions, as well as primary and recurrent tumors from seven patients with p16-negative HNSCC.

**Methods:**

We performed targeted panel Next Generation Sequencing (161 genes) to analyze somatic variants from sequentially collected, matched formalin-fixed paraffin-embedded tissue (normal, premalignant, HNSCC) from two patients. These samples plus samples from five additional patients were analyzed with the Nanostring PanCancer Immune Panel. In addition, we performed shallow whole genome sequencing (0.5x coverage on average) on samples from three of these patients. Patients were, apart from one case, primarily treated with curative-intent surgery, and received subsequent adjuvant treatment, if indicated.

**Results:**

The most frequently mutated genes were *TP53* and *NOTCH1*. Other mutated genes included *NOTCH3* and *CDKN2A*, among others. A significant number of mutations were private to dysplasia and invasive carcinoma, respectively, however, almost 20% were shared between them. Increasing genomic instability was observed when comparing histologically normal squamous mucosa with higher levels of dysplasia. High-grade dysplasia showed similarly rearranged genomes as invasive carcinoma. Pathways related to interferon alpha and gamma response were upregulated even in moderate dysplastic lesions with increasing expression in higher grades of dysplasia and carcinoma. *SPINK5*, a known tumor suppressor gene in HNSCC, was already downregulated in low-grade dysplastic lesions, indicating an early deactivation in the evolution of the disease.

**Conclusion:**

Genomic alterations as well as aberrant immune gene expression can be observed early in the evolution of tumors of the upper aerodigestive tract, highlighting the potential for targeting early mechanisms of disease progression.

## 1 Introduction

Head and neck squamous cell carcinoma (HNSCC) is a heterogeneous disease that may develop in large carcinogen-induced premalignant fields of genetically altered cells. Field cancerization was first observed and described by Slaughter et al. in 1953 (1). At that time, the investigators concluded that the mucosa of the upper aerodigestive tract had undergone changes which were likely due to continued carcinogen exposure and could therefore become more susceptible to the development of multiple foci of malignant transformation (2). At its core, HNSCC evolution involves a multifaceted interplay of genetic mutations and interactions with the local microenvironment. It is well established that the process of tumor evolution progresses through stages of premalignancy wherein cells acquire abnormal geno- and phenotypes that increase their propensity for malignant transformation. Clonally unrelated premalignant cells in a cancerized field can often be found distant from each other and contribute to local disease recurrence or the development of a second primary tumor in the upper aerodigestive tract (3–9). However, the exact order and timing of these genetic changes remains to be determined for HNSCC pathogenesis. Frequent alterations in gene copy number, for example, are a hallmark of advanced cancers (10). Normal epithelium on the other hand displays very rarely copy number alterations (11) but may instead harbor numerous somatic mutations (12–14). Much less is known about the process of transformation from normal epithelium to premalignant lesions and subsequent evolution into malignant tumors. Evidence suggests that copy number alterations may play a key role in driving early malignancy (11, 15, 16). This concept adds another layer of complexity to the understanding of how malignancies develop in the upper aerodigestive tract and is particularly significant in the context of HNSCC, as it underscores the importance of not only treating the primary tumor but also monitoring and addressing potential premalignant changes in the surrounding tissue. Understanding the biology of premalignancy is therefore a critical enabler to developing the right approaches to intercepting the disease. Yet knowing which individuals with premalignancies will progress and develop clinically significant cancers during their lifetimes remains one of the most important challenges of reducing HNSCC morbidity and mortality. Simultaneously, it has become increasingly evident that the progression of tumors and the immune responses are profoundly interconnected, extending beyond genetic mutations and cellular transformations (17–19). Recent advancements in immune checkpoint therapy have demonstrated improved overall survival in a proportion of patients with recurrent/metastatic HNSCC (20–24). Given the impact of immunotherapy in advanced disease, modulation of the immune microenvironment might prove beneficial as a prevention strategy in patients with high-risk premalignant mucosal lesions. Available data indicates that immune surveillance may play an important role in preventing the transition of premalignancy to cancer (25–27). However, the extent to which an active immune system influences the genome evolution of a tumor (or vice versa) remains unknown, particularly with respect to the extensive genomic and transcriptomic heterogeneity within a tumor and its surrounding microenvironment (28, 29).

Here, we retrospectively analyzed archived FFPE tissue samples from primary and recurrent tumors as well as matched premalignant and normal squamous mucosa from patients with p16-negative HNSCC. We used targeted panel Next Generation Sequencing (NGS) and shallow whole genome sequencing (WGS) on a selected number of tissues to profile the mutational landscape of multi-regionally and sequentially collected samples. Additional patients’ samples were analyzed with the Nanostring PanCancer Immune Panel to explore the mechanisms underlying immune evasion. The aims of this study were to provide a characterization of the preconditioned squamous mucosa in a cancerized field and their corresponding primary and, if available, recurrent tumors to shed light into their genomic, immune microenvironmental and evolutionary relationship. We confirm that genomic alterations, along with dysregulation in immune-related gene expression, are detectable at an early stage in the development of upper aerodigestive tract tumors. These observations underscore the potential for targeting the mechanisms involved in the early stages of disease progression, offering new avenues for therapeutic intervention.

## 2 Materials and methods

### 2.1 Case collection

A retrospective cohort study was conducted to profile nucleic acids from resected tumor tissue, dysplastic lesions, and normal squamous mucosa. All samples were previously obtained and archived from a cohort of seven patients with HNSCC and/or dysplastic lesions in the upper aerodigestive tract. Patients with p16-negative HNSCC and/or dysplastic lesions of the oral cavity, pharynx, or larynx were included. Patients with distant metastasis (cM1) or other active malignancies as well as p16-positive cases were excluded. All patients were staged to exclude distant metastasis with computed tomography (CT) and/or magnetic resonance imaging (MRI). Patients with confirmed HNSCC received primary surgery with curative intent and adjuvant radio(chemo)therapy, or primary radiochemotherapy, according to the National Comprehensive Cancer Network guidelines (NCCN Guidelines) (30) and following recommendation by the local multidisciplinary tumor board. Immunohistochemical staining for p16 was done as part of the routine histopathological work-up.

### 2.2 Nucleic acid extraction

Formalin-fixed paraffin-embedded (FFPE) tumor blocks of the resected specimen, if available, as well as corresponding dysplastic lesions and normal squamous mucosa were obtained from the archives. Sections from FFPE tumor tissue samples were prepared followed by hematoxylin-eosin (H&E) staining of one slide. Tumor areas were microdissected from subsequent unstained sections and used for nucleic acid preparation. For dysplastic lesion and normal squamous mucosa analysis, twenty sections were cut including three slides for H&E staining (first, middle and last slides) and the residual ones for further analysis. The final histopathological status was confirmed by an experienced head and neck pathologist. DNA was extracted from the FFPE tissue samples using the GeneRead DNA FFPE Kit (Qiagen, Hilden, Germany). RNA was extracted from the FFPE tissue samples using the RNeasy FFPE Kit (Qiagen, Hilden, Germany). Nucleic acid concentrations were measured using a Qubit 2.0 fluorometer (Invitrogen, CA, USA) and the Qubit dsDNA HS (High Sensitivity) Assay Kit (Thermofisher, MA, USA).

Ten ml blood was collected into K2 EDTA tubes (BD Biosciences) and processed to buffy coat, i.e. leukocytes, for extraction of germline DNA, respectively, as described previously (31).

### 2.3 Sequencing library preparation, Next Generation Sequencing and variant interpretation

Library preparation with the AmpliSeq for Illumina Oncomine Comprehensive Assay v3 (Illumina, CA, USA), targeting 161 cancer-associated genes (**Supplementary Table 1**), subsequent sequencing, and variant calling were performed as described in detail previously (31). Briefly, sequencing was performed on an Illumina NextSeq 500 system using NextSeq 500/550 High Output Kits v2.5 according to the manufacturer’s protocols. Analysis of the results was performed with the Illumina Local Run Manager, subsequent annotation of VCF-files using wANNOVAR (32) and an in-house python script filtering for relevant mutations. Sequencing quality parameters are shown in **Supplementary Table 2**. Alterations were confirmed with the Integrative Genomics Viewer (IGV, Broad Institute, MA, USA). Variants were judged as relevant based on the interpretation criteria utilized in ClinVar (33). Pathogenicity prediction algorithms and other publicly available databases were used for variant interpretation (VarSome (34), dbSNP (Available from: https://www.ncbi.nlm.nih.gov/snp/)). Only likely pathogenic and pathogenic mutations as well as VUS (variant of unknown significance or not evaluated in ClinVar with a prediction trend of being likely pathogenic) were reported (**Supplementary Table 3**). Single-nucleotide variants (SNV), multi-nucleotide variants (MNV), small insertions, deletions, indels, and copy number variation (CNV) were analyzed.

### 2.4 Shallow whole genome sequencing and copy number calling

Sequencing libraries were prepared using 4-60ng DNA by the ThruPLEX Tag-Seq Kit (Takara Bio, France). Quantity and quality of the libraries were assessed by Qubit dsDNA High Sensitivity Assay Kit (Thermofisher, MA, USA). Sequencing libraries were pooled at equal amounts. WGS at an average coverage of 0.5x was performed on Illumina NexSeq 500 using 2 x 150 bp paired-end sequencing. A normal reference was created from shallow WGS of leukocyte-derived genomic DNA of the respective patients as well as a panel of healthy donors sequenced at 0.5x coverage. High-quality and nonduplicate reads were aligned to human reference genome GRCh38 using BWA (35). Aligned reads were pre-processed, removing unreliable or badly mapped reads, and excluding PCR artifacts. Reads were corrected for GC content. Copy number analysis was performed using GATK (http://software.broadinstitute.org/gatk/) and the ichorCNA software (36) for tumor fraction and ploidy estimation.

### 2.5 Gene expression profiling

We performed gene expression profiling on 36 sequentially collected RNA samples from seven patients using the nCounter PanCancer Immune Profiling Panel (NanoString Technologies, U.S.A.), targeting 770 transcripts including up to 40 reference genes (**Supplementary Table 4**). 150 ng RNA was used for gene expression analysis according to the manufacturer’s instructions. Data analysis was performed using the nSolver 4.0 software (https://www.nanostring.com/products/analysis-software/nsolver). The geometric mean of the counts relative to each sample, the mean plus two standard deviations and the total sum of counts were used to correct the data for technical, background, and batch effects, respectively. The expression profiles of housekeeping genes and quantile normalization were used to account for inter-sample variations within the panel (**Supplementary Table 5**). Heatmaps of the most significantly, differentially expressed genes using log2 fold change of normalized gene expression values were generated using ClustVis (37). Differentially expressed gene analysis was performed using the Gene Set Enrichment Analysis (GSEA) v4.3.2 software (38). Significant p-values and false discovery rates (FDR) were obtained. The criteria for differentially expressed genes have been set as FDR<0.25. The thresholds were set to log2 fold change > 1 and adjusted p-value < 0.05 for further analysis. Lists of ranked genes in differentially expressed signaling pathways are presented in **Supplementary Table 6**. **Supplementary Tables 7–9** show the most significantly upregulated signaling pathways (c6 oncogenic pathways, c7 immune pathways, hallmark pathways) for each phenotype.

## 3 Results

### 3.1 Patient characteristics

**Figure 1:**
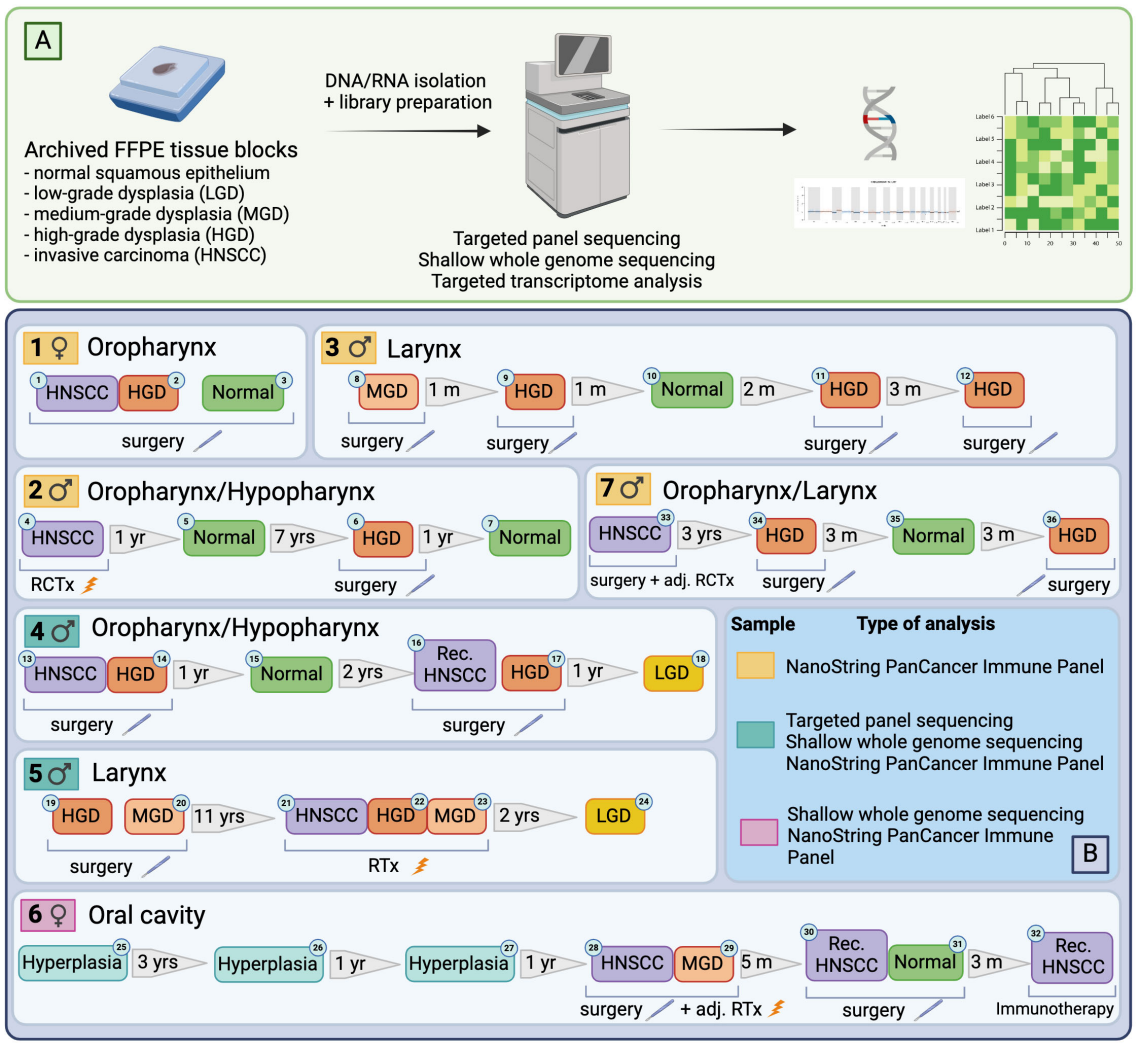
Illustration of the workflow **(A)** and case selection **(B)**. Figure generated with BioRender.com.

Seven patients who were diagnosed with a p16-negative HNSCC and/or premalignant lesion(s) of the upper aerodigestive tract (low-/moderate/high-grade dysplasia) and primarily treated by surgery or radiochemotherapy were included into this study. Most patients (71%) were male, and all had a diagnosis of invasive squamous cell carcinoma at some point during their treatment. Apart from one patient, all were scheduled for curative-intent surgery and, if applicable, received adjuvant treatment according to the recommendations of the multidisciplinary tumor board. Patients were followed up and received multiple resections or biopsies, respectively, throughout their treatment. 4/7 patients developed a histologically confirmed recurrence after completing their treatment. A total of 36 retrospectively collected primary tumor, premalignant and normal mucosal tissue samples from seven patients were selected for further analysis. A schematic overview of the patient cohort, including types of analyses used on each sample, is shown in **Figure 1**.

### 3.2 Patient summaries

Each patient is described in brief below and in more detail in the Supplementary Material (**Supplementary Table 10**).

Patient 1 was an 81 y/o female smoker who received surgical treatment for a pT1 pN0 squamous cell carcinoma (SCC) of the soft palate with a surrounding field of high-grade dysplasia which had been confirmed by multiple biopsies.

Patient 2 was a 72 y/o male smoker who initially received primary radiochemotherapy for a cT3 cN2b hypopharyngeal carcinoma. This was followed by multiple resections of dysplastic lesions at the left glossotonsillar sulcus until eventually a large cT4a cN2b carcinoma of the base of the tongue extending into the left lateral tongue was diagnosed 9 years later. The patient received palliative radiotherapy.

Patient 3 was an 87 y/o male smoker who presented with dysphonia and initial biopsy of the right vocal cord showed a moderate dysplastic lesion. Control panendoscopies with multiple biopsies taken confirmed different grades of dysplasia on both vocal cords until eventually 7 months later an invasive cT1a laryngeal SCC was confirmed histologically and subsequently resected.

Patient 4 was a 54y/o male smoker who received surgical treatment for an invasive pT2 pN0 hypopharyngeal carcinoma (right sinus piriformis) on a background of a high-grade dysplasia at age 54. Following initial tumor resection and a bilateral neck dissection, a high-grade dysplastic lesion at the right glossotonsillar sulcus was subsequently histologically confirmed and resected 8 months later, and at the right aryepiglottic fold 2 years later. Another 7 months later a pT1 carcinoma of the base of the tongue was confirmed and treated by transoral laser microsurgery. In addition, high-grade dysplastic lesions at the posterior wall of the pharynx were resected. The patient presented with a rpT3 rpN1 hypopharyngeal carcinoma 12 months later. Following a pharyngo-laryngectomy, bilateral node picking and reconstruction using a pectoralis major flap, the patient subsequently received adjuvant radiotherapy.

Patient 5 was a 57y/o male smoker who was surgically treated for a high-grade dysplasia of the right vocal cord and 4, 7, and 11 years later for a high-grade dysplasia of the left vocal cord. Three years following the previous resection, the patient was diagnosed with an invasive cT1b laryngeal carcinoma on the background of a high-grade dysplasia for which he received primary radiotherapy. Sixteen months later a pT1 carcinoma of the epiglottis was treated by transoral laser resection. The patient received a laryngectomy 18 months later due to a local recurrence with extensive dysplasia throughout the larynx. Another local recurrence on the background of the cancerized field as well as a pulmonary metastasis were diagnosed 12 months later, and systemic treatment was initiated.

Patient 6 was a 62y/o female non-smoker who had multiple biopsies of the left lateral tongue within 3- and 4-years following diagnosis of a lateral tongue lesion that showed hyperplastic mucosa without evidence of dysplasia or invasive carcinoma. A pT3 pN0 carcinoma of the left lateral tongue was confirmed 5 years later and the patient received a transoral tumor resection and bilateral neck dissection and subsequent adjuvant radiotherapy. A local recurrence yrpT3 was confirmed 5 months later and treated by surgical resection. Only 3 months later the patient presented with an extensive loco-regional as well as distant recurrence that was unresectable and the patient was subsequently started on systemic treatment.

Patient 7 was a 68y/o male smoker who received surgical treatment for a pT3 pN3b oropharyngeal carcinoma (left base of tongue and glossotonsillar sulcus) and subsequent adjuvant radiochemotherapy. A high-grade dysplasia was confirmed at the larynx (arytenoid cartilages) 3 years later for which the patient received photodynamic therapy multiple times. Several biopsies confirmed extensive dysplasia throughout the larynx and pharynx.

### 3.3 Point mutations and copy number changes in primary tumors, premalignant lesions, and normal mucosa

**Figure 2:**
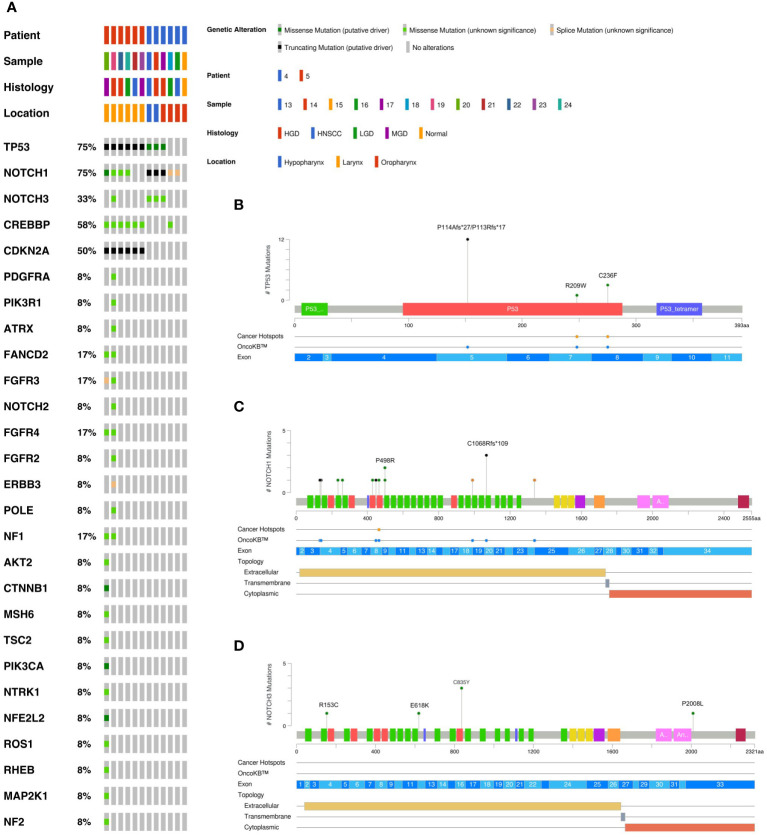
Overview of somatic variants identified in HNSCC, HGD, MGD, LGD, hyperplasia and normal tissue from patients 4 and 5. **(A)** Oncoplot of the most frequently mutated genes. This shows a list of genes arranged based on the total number of variants in each gene with the percentage representing the ratio of samples with its genetic alteration to the total number of samples. Type of mutation, sample histology and anatomical localization are explained in the legend. The variant prevalence and spectrum of *TP53* **(B)**, *NOTCH1* **(C)**, and *NOTCH3* **(D)** genes in this cohort. Green circles indicate missense mutations, black circles truncating mutations, and orange circles splice sites. HNSCC, Head and Neck Squamous Cell Carcinoma; HGD, high grade dysplasia; MGD, medium grade dysplasia; LGD, low grade dysplasia.

DNA was isolated from sequentially collected, matched FFPE tissue (normal squamous mucosa, premalignant mucosal lesions, HNSCC). Pathological review ensured the correct histological diagnosis before proceeding with the analysis. We performed targeted panel NGS (161 genes) to analyze somatic variants from 12 samples that were obtained from two patients (patient 4 and patient 5). DNA sequencing was successful in all samples with a mean DNA sequencing depth of 3670.4. The average identified number of somatic variants per tumor after applying our filtering criteria was 3.3 (range 1 – 5). For dysplastic lesions we identified on average 8.375 somatic mutations (range 3-24). No somatic mutations were identified in normal squamous mucosa (sample 15) after applying our filtering criteria. The most frequently mutated genes were *TP53* (9/12) with predominantly missense and truncating mutations (**Table 1**) and NOTCH1 (9/12) with missense and truncating mutations as well as splicing variants (**Table 2**) identified (**Figure 2**). Other mutated genes included *CDKN2A* (6/12), *CREBBP* (7/12) and *NOTCH3* (4/12). *CTNNB1*, *PDGFRA*, *PIK3R1*, *FGFR3*, *FGFR4*, *NF1*, *MSH6, ATRX, FANCD2, TSC2, PIK3CA*, and *AKT2*, among others, were each mutated in one or two samples. Many variants at low variant allele fraction (VAF) were identified in samples 19 and 20 and are likely due to extensive field cancerization (**Supplementary Table 3**). In total 43/53 variants were private to dysplastic lesions and not detected in invasive carcinoma whereas only 2/53 variants were private to invasive carcinoma and not detected in dysplastic lesions. 8/53 variants were found in both dysplasia as well as invasive carcinoma. The median VAF of all detected somatic variants was 3.4% (range 1.7% – 95.2%). The median VAF of *TP53* in all tumors was 28% (range 22.9 – 83.0) and of all dysplastic lesions 9.8% (range 1.8 – 47.5), respectively. For *NOTCH1*, the median VAF in all tumors was 3.7% (range 2.6 – 25.8) and of all dysplastic lesions 2.7% (range 1.7 – 35.3), respectively.

**Table 1:** Overview of somatic variants identified in TP53 in HNSCC, HGD, MGD, LGD, hyperplasia and normal tissue from patients 4 and 5. HNSCC, Head and Neck Squamous Cell Carcinoma; HGD, high grade dysplasia; MGD, medium grade dysplasia; LGD, low grade dysplasia; VAF, variant allele frequency.

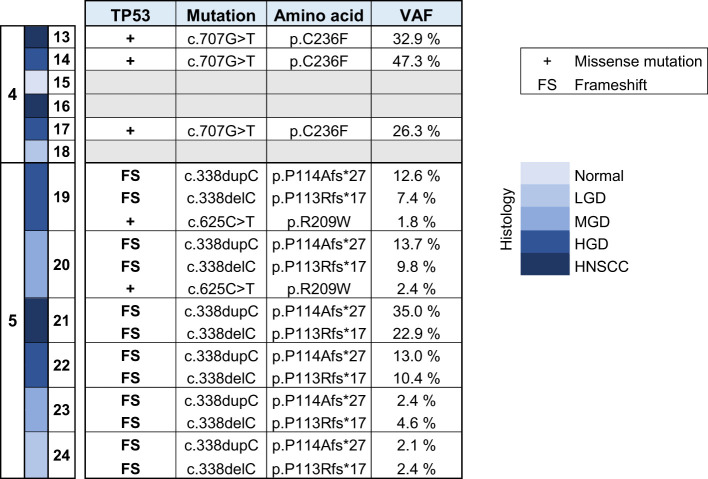

**Table 2:** Overview of somatic variants identified in NOTCH 1 and NOTCH 3 in HNSCC, HGD, MGD, LGD, hyperplasia and normal tissue from patients 4 and 5. HNSCC, Head and Neck Squamous Cell Carcinoma; HGD, high grade dysplasia; MGD, medium grade dysplasia; LGD, low grade dysplasia; VAF, variant allele frequency.

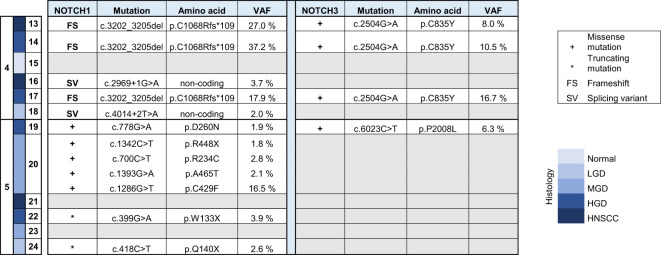

**Figure 3:**
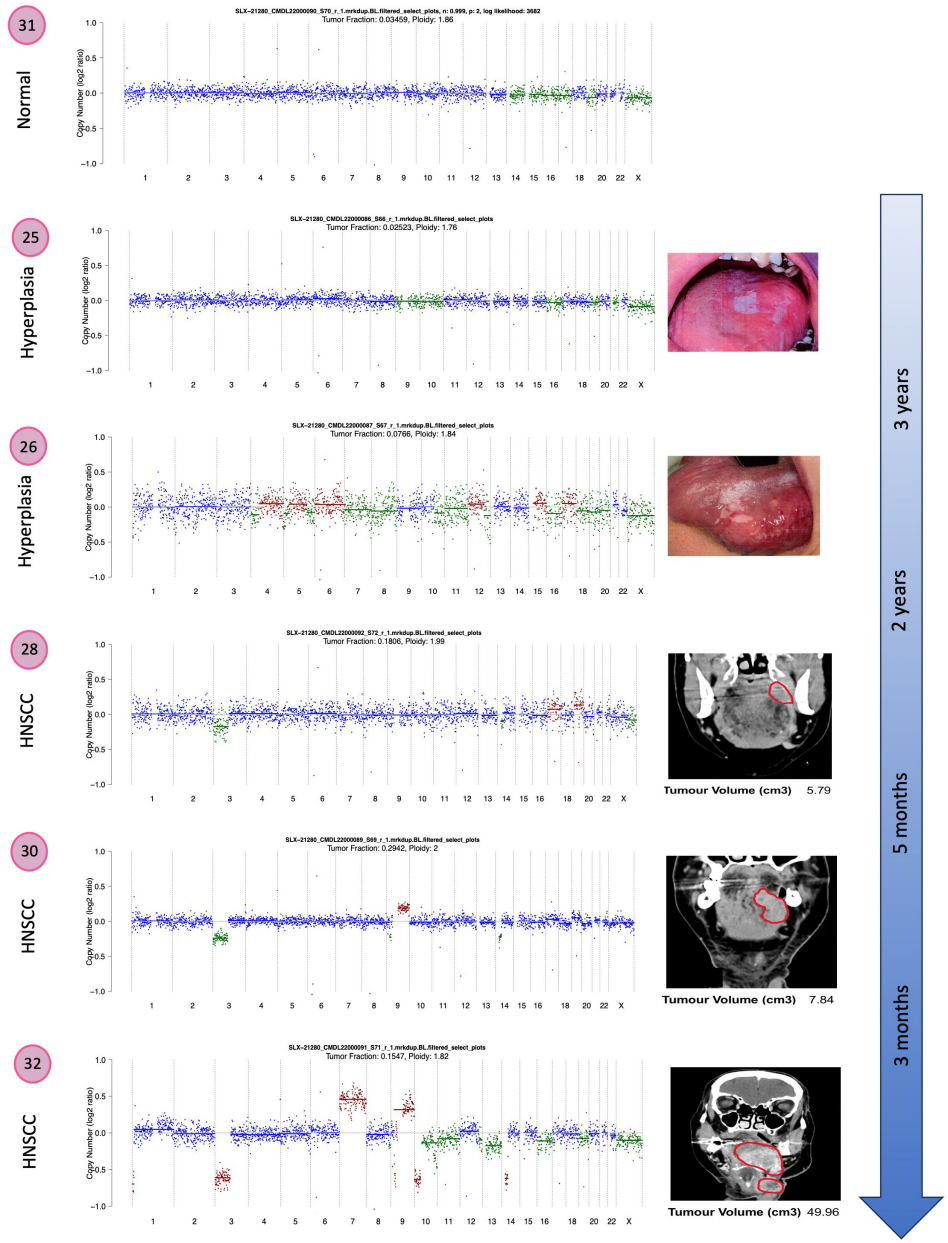
Copy number plots from normal mucosa (31), hyperplasia (25, 26) and HNSCC (28) with 2 recurrences (30, 32) from the same patient (case 6). Corresponding images of the tongue lesions as well as CT images with the tumor circled in red are shown on the right. Tumor volume is given in cm^3^. Diploid copies are shown in blue, gains in red and losses in green.

In addition to targeted panel sequencing, we performed shallow WGS (0.5x coverage on average) on 20 samples from three patients (patients 4, 5 and 6). For case 6, increasing genomic instability could be observed when comparing histologically normal squamous mucosa (sample 31) with hyperplastic mucosa (sample 26) and primary and recurrent tumors of the lateral tongue (samples 28, 30 and 32). The latter displayed an increasing loss of chromosome arm 3p over time (**Figure 3**). High-grade dysplastic samples (samples 14 and 17) from case 4 had similarly rearranged genomes as was seen in the HNSCC sample (sample 13) (**Supplementary Figure 1**). Sample 16 was likely contaminated by normal tissue and has therefore been disregarded in this analysis. Low-grade dysplasia (sample 18) was characterized by an equally quiet genome as normal squamous mucosa (sample 15) (**Supplementary Figure 1**). For case 5, moderate dysplasia (samples 20 and 23) displayed several copy number changes more similar to high-grade dysplastic samples (samples 19 and 22) than compared to low-grade dysplasia (sample 24). Sample 21 illustrated the characteristically highly rearranged genome of an invasive carcinoma (**Supplementary Figure 2**).

### 3.4 Differential gene expression analysis in primary tumors, premalignant lesions, and normal mucosa

**Figure 4:**
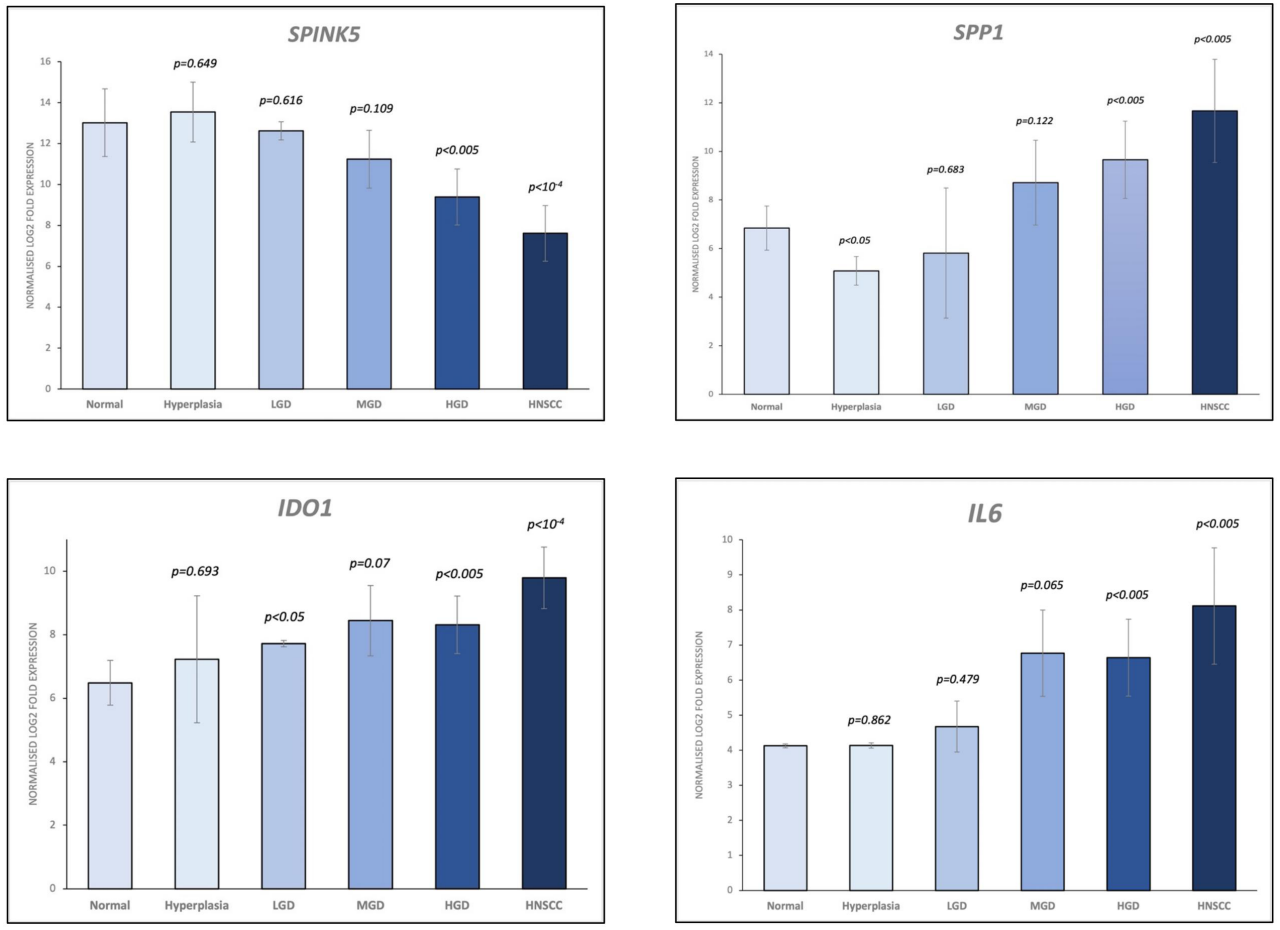
Selection of the top 4 most significantly (p<0.05), differentially expressed genes which showed a stepwise increase/decrease from benign to malignant phenotype. Gene expression values are shown as log2 fold change in expression. HNSCC, Head and Neck Squamous Cell Carcinoma; HGD, high grade dysplasia; MGD, medium grade dysplasia; LGD, low grade dysplasia.

**Figure 5:**
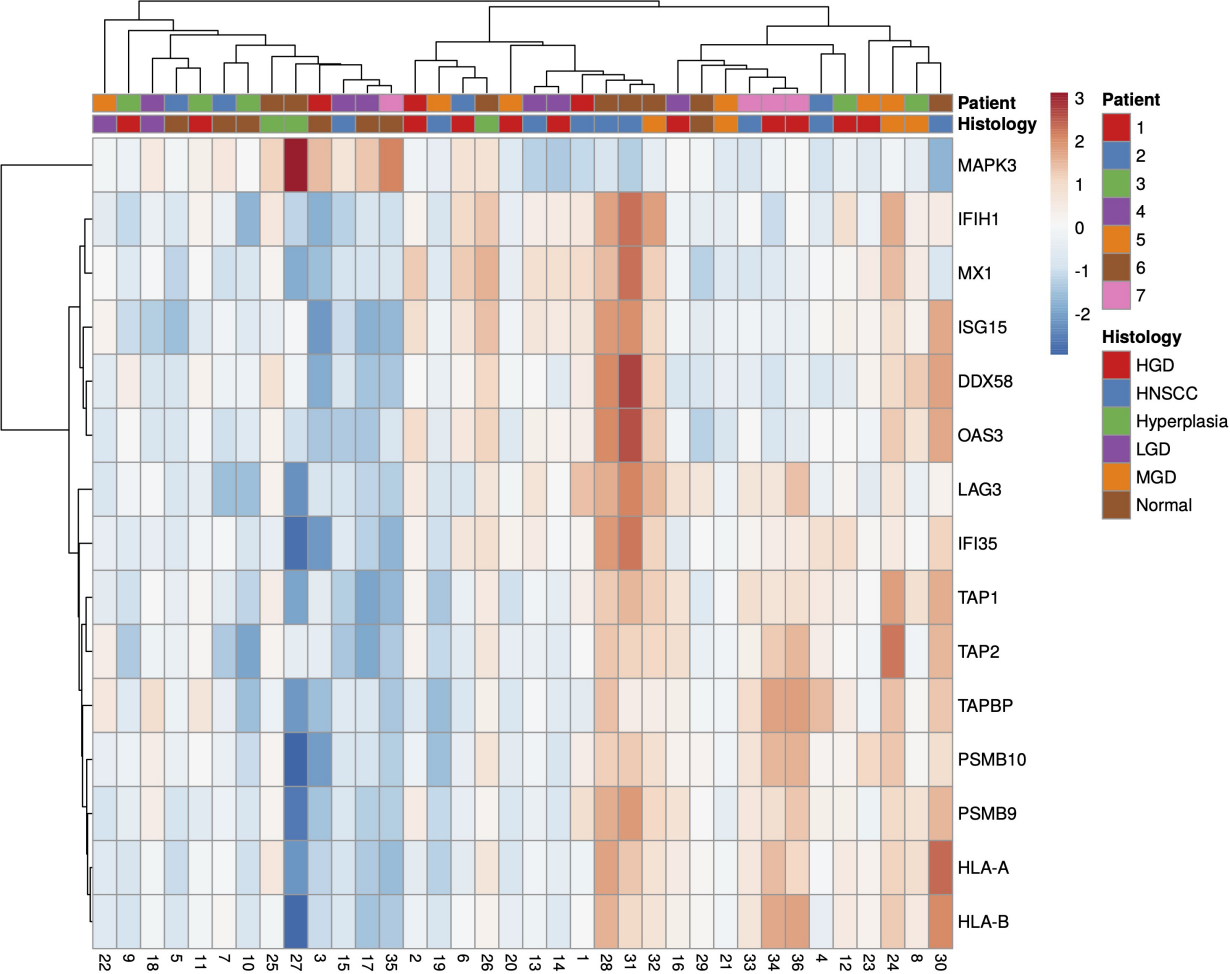
Heatmap of all significantly (p<0.05), differentially expressed genes in HNSCC, HGD and MGD across all samples, using log2 fold change of normalised gene expression values. Samples are listed on the horizontal axis and genes are listed vertically. Red/orange indicates high scores; blue indicates low scores. HNSCC, Head and Neck Squamous Cell Carcinoma; HGD, high grade dysplasia; MGD, medium grade dysplasia; LGD, low grade dysplasia.

**Figure 6:**
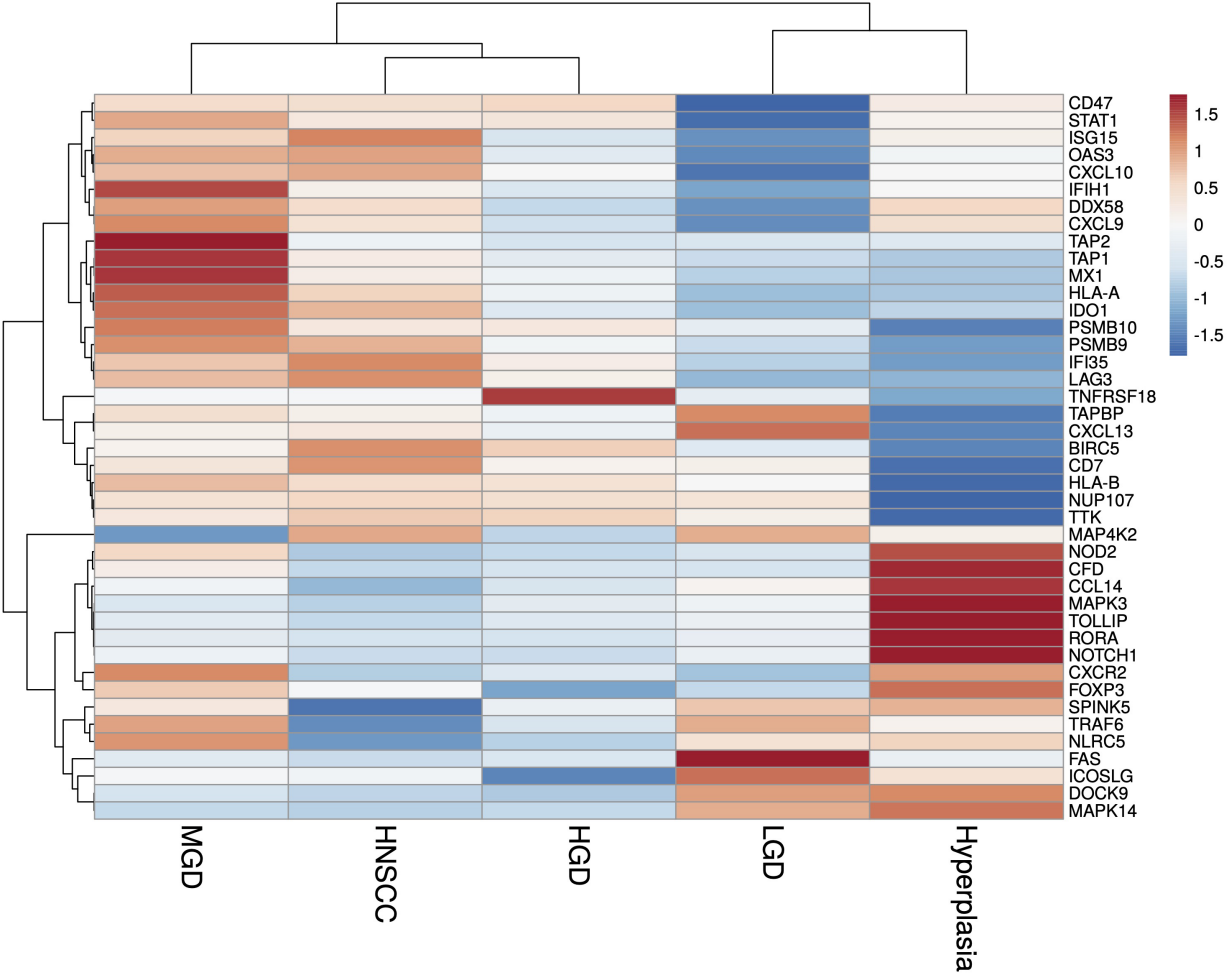
Heatmap of the most significantly (p<0.05), differentially expressed genes identified in HNSCC and HGD across grouped samples, using log2 fold change of normalised gene expression values. Samples are listed on the horizontal axis and genes are listed vertically. Red/orange indicates high scores; blue indicates low scores. HNSCC, Head and Neck Squamous Cell Carcinoma; HGD, high grade dysplasia; MGD, medium grade dysplasia; LGD, low grade dysplasia.

**Figure 7:**
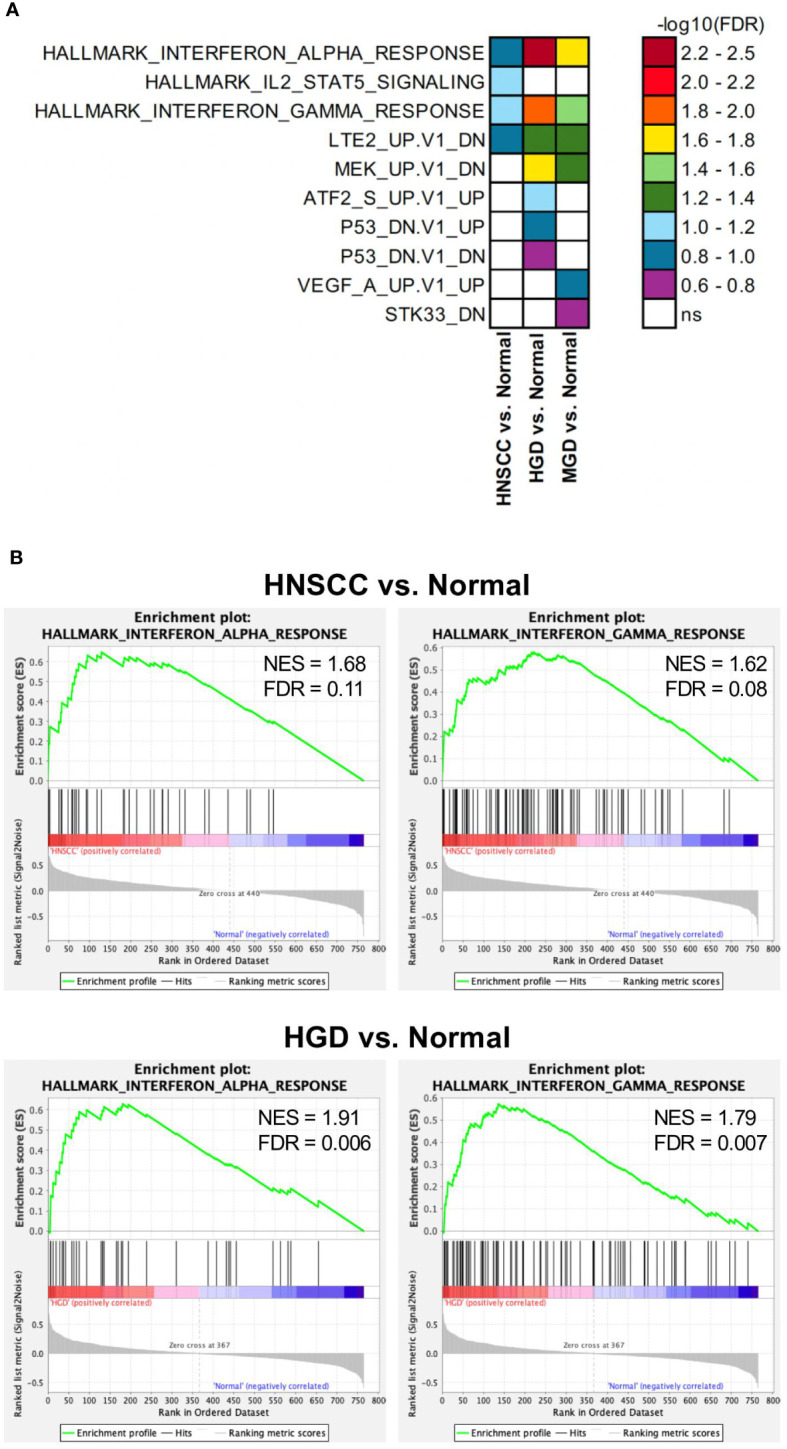
Differentially regulated pathways in HNSCC vs. Normal, HGD vs. Normal and MGD vs. Normal. GSEA results from analyses with Hallmark, c6 oncogenic signature and c7 immune pathway data sets. **(A)** Significantly enriched gene sets (FDR<0.25) from the Hallmark and c6 oncogenic signature data sets in indicated comparisons are depicted. FDR are presented as -log_10_. **(B)** GSEA results of the Hallmark Interferon alpha response and gamma response gene sets comparing the indicated groups. GSEA, Gene Set Enrichment Analysis; FDR, false discovery rate; ns, not significant; HNSCC, Head and Neck Squamous Cell Carcinoma; HGD, high grade dysplasia; MGD, medium grade dysplasia.

A total of 36 samples representing various histological states of premalignancy and malignancy from seven patients were analyzed for alterations in gene expression in oncogenic immune signaling pathways. We examined gene expression patterns comparing all seven cases by selecting the most significantly, differentially expressed genes which showed a stepwise increase or decrease from benign to malignant phenotype and were observed in more than one patient (**Supplementary Figure 3**). Four significantly, differentially expressed genes are depicted in **Figure 4**. The highest ranked transcript was *SPINK1* which was consistently downregulated in all cases with decreasing expression from low-grade (-1.03-fold, p = 0.616) to moderate (-1.16-fold, p = 0.109) to high-grade dysplasia (-1.39-fold, p < 0.005), and lowest expression in HNSCC (-1.71-fold, p < 10^-4^). Another transcript that showed a stepwise differential expression in three cases (cases 4, 5, and 6) was *SPP1*. Lowest expression of *SPP1* was observed in hyperplastic samples, with increasing expression from low-grade (0.79-fold, p = 0.76) to high-grade dysplasia (1.24-fold, p < 10^-4^) and highest expression seen in invasive carcinoma (1.6-fold, p < 10^-5^). Other differentially expressed transcripts include *S100A7*, *LCN2, CXCL14*, and *CD207* (decreasing expression from premalignant lesions to invasive carcinoma), as well as *IDO1*, *IL6*, *IL8*, *THBS1*, and *FN1* (increasing expression from premalignant lesions to invasive carcinoma). Looking across all samples, other candidates that were among the top differentially expressed genes in moderate dysplasia, high-grade dysplasia, and HNSCC included *LAG3*, *PSMB9*, and *PSMB10*, among others (**Figure 5**). When investigating the top scoring 15 differentially expressed genes among different histological groups, we observed a clustering of hyperplastic with low-grade dysplastic samples and a clustering of moderate with high-grade dysplastic as well as invasive samples (**Figure 6**). Among the top downregulated genes with increasing malignancy were *SPINK5, MAPK3*, and *MAPK14. LAG3, IFI35*, *IDO1*, and *CD7* were among those genes with increasing expression from hyperplasia to HNSCC. We then applied GSEA to identify functional gene sets that were correlated with increasing levels of malignancy. The most significantly upregulated signaling pathways were the interferon gamma and alpha response pathway (**Figure 7** and **Supplementary Table 11**). Interestingly, an upregulation of these pathways could already be seen in moderate dysplasia. In HNSCC, IL2 STAT5 signaling was significantly enhanced (**Figure 7** and **Supplementary Figure 4**). Compared with LGD and hyperplasia samples, an upregulation of TNFα signaling via NFκB was observed in moderate and high-grade dysplasia (**Supplementary Figure 5**).

## 3 Discussion

In this study, we have conducted a focused analysis of the genome and immune microenvironment from multiple, matched normal squamous tissues, premalignant lesions, primary, and recurrent tumors from seven patients with p16-negative HNSCC with the aim to identify novel molecular mechanisms that could drive tumor evolution in the upper aerodigestive tract. The unique aspect of this study is the comprehensive profiling of multi-regionally and sequentially collected and well annotated samples at different disease stages, which also included a focus on the role of the immune microenvironment.

Targeted analysis of genetic alterations revealed a characteristic mutational pattern in HNSCC, akin to findings reported in previous studies (39–41). The most prevalent genetic mutations observed in our cohort were in the *TP53* and *NOTCH1* genes, followed by mutations in *CDKN2A, CREBBP, NOTCH3, CTNNB1, FGFR3*, and *FGFR4*, among others. A notable proportion of mutations were exclusive to dysplasia and carcinoma, respectively, although approximately 20% were shared between these two stages. Gene mutations rated as (likely) pathogenic that were already identified in low-grade and moderate dysplastic samples included inactivating mutations in the tumor suppressor genes *TP53*, *CDKN2A*, and *NOTCH1*, implying a crucial role in stepwise carcinogenesis towards HNSCC. These results corroborate the empirical progression model of Califano et al., where *TP53* is disrupted early (5, 42). In that model, the earliest event of disease progression includes loss of chromosome arm 9p, which harbors *CDKN2A* (43), progressing to loss of chromosome arms 17p, where *TP53* resides, and 3p. In our cohort, in earlier grades of dysplasia, individual genes were present at low prevalence (<15% VAF). This may support an early role in tumorigenesis as suggested by Leshchiner et al. who inferred genetic progression from exome sequencing of primary HNSCC tumors (44). While the role of *TP53* as tumor suppressor gene in HNSCC is well established and different mutations could be linked to increased malignancy (45) and therapy-resistance (46), the functional significance of *NOTCH1* mutations is less clear (47). *NOTCH1* and *NOTCH3* belong to the family of cell surface receptors that transduce juxtacrine signals of delta-like canonical Notch ligands and jagged canonical Notch ligands from adjacent cells, with significant roles in directing tissue commitment and cell differentiation (48). Loss of function of *NOTCH1* has been linked to the acquisition of stem-like properties and a more aggressive phenotype. However, tumor-suppressing mechanisms of *NOTCH1* signaling are not entirely understood (49). High expression of Notch3 on the other hand has been linked to poorer prognosis in SCC (50). Observed loss of 9p and therefore *CDKN2A* with subsequent cell cycle dysregulation as an early event in the carcinogenesis are in line with previous findings (51). The function of tumor suppressors is inherently difficult to restore. However, a recent study could show meaningful response rates to selective CDK4/6 inhibitors in *CDKN2A*-altered HNSCC, proving the clinical significance of *CDKN2A* mutations (52). Whether this approach could also target premalignant lesions, needs to be established. Recent work has demonstrated a role for chromosomal instability driving selection in cancer evolution with early somatic copy number alterations potentially being implicated in tumorigenesis (53). Our results also support an increasing genomic instability over time. Case 5 showed a high rate of chromosomal alterations even in moderate dysplasia, including loss of 3p and 9p, that was followed by multiple disease recurrences. Similarly, case 6 demonstrated progressive loss of 3p with subsequent local tumor recurrences. Previous studies have shown that dysplastic lesions that harbor 3p loss are 33 times more likely to progress to invasive carcinomas than those without (54) and that 3p deletion may be associated with poor disease outcomes (55). Furthermore, high-grade dysplasia showed a similarly rearranged genome as was seen in HNSCC, confirming its behavior like an invasive carcinoma. The existence of these multilocular distinct premalignant conditions therefore presents the need and opportunity to define which are at greatest risk for progression, including if, how, and when to intervene.

A recent comprehensive study on early-stage untreated lung cancers from the TRACERx cohort demonstrated an interplay between the immune and genomic landscapes through copy number loss as a mechanism of subclonal immunoediting. Immunoediting mechanisms may impact on tumor evolution, affecting either antigen presentation or neo-antigenic mutations both at DNA and RNA level with chromosomal instability driving loss of neo-antigens (28). Other studies were able to establish a link between somatic copy number alterations and markers of immune evasion, with highly aneuploid tumors being less responsive to immunotherapy (56). In HNSCC, cumulative loss of chromosome arm 9p has been shown to be the strongest driver of immune evasion. Simultaneously, copy number-defined high-risk oral preinvasive and early invasive lesions were immunogenic, suggesting a possible clinical benefit of augmenting immune surveillance (57). Nevertheless, the exact role of the immune microenvironment in the early stages of cancer development remains largely unknown, and particularly how survival and clonal expansion of premalignant cells may be fostered by an immunosuppressive microenvironment that hinders immune surveillance remains elusive. In our study, pathways associated with the interferon alpha and gamma response exhibited heightened activity, even in moderate dysplastic lesions, with a progressively amplified expression in higher dysplasia grades and in carcinoma. Interferon gamma has been linked to antitumor-immune response and increased interferon gamma signaling has been proposed as a prediction marker for response to immune checkpoint therapy in HNSCC (58). In contrast, low-dose interferon gamma might lead to tumor progression (59). Early upregulation of this signaling pathway in dysplasia might indicate an increased, yet insufficient, immune response. Likewise, interferon alpha signaling contributes to antitumor activity in early stages and has been shown to promote tumor growth in equilibrium and escape phase of cancer development (60). It can therefore be speculated, whether immunomodulatory therapy targeting these pathways in dysplasia (in combination with immune checkpoint therapy) could have beneficial effects. Interestingly, a recent study could show that immune checkpoint inhibition can achieve regression in high-risk oral leukoplakia (61). Moreover, progression to invasive carcinoma could be linked to 9p21.3 loss, encompassing the type I interferon gene cluster and *CDKN2A*, pointing towards a crucial role in therapy resistance and carcinogenesis.

Single gene expression analysis revealed a few markers differentially expressed in different grades of dysplasia compared to HNSCC. One candidate gene that was upregulated in moderate dysplastic compared to low-grade dysplastic samples with highest expression in HNSCC was secreted phosphoprotein 1 *(SPP1)*, also known as osteopontin (*OPN*). *SPP1*, an integrin-binding glycophosphoprotein, has been shown to be associated with immune cell infiltration and found to be upregulated in multiple cancers, including HNSCC (62). High *SPP1* expression has previously been shown to be associated with poor overall survival in colorectal cancer, with increasing expression from normal tissue to primary tumors and ultimately to metastases (63). Indoleamine 2,3-dioxygenase 1 (*IDO1*), another significantly differentially expressed gene that we found to be upregulated from benign to malignant phenotype, is a rate-limiting enzyme in the conversion of the essential amino acid tryptophan to kynurenine. *IDO1* is overexpressed in various tumor types and associated with worse overall survival (64). It has been shown to be involved in immunosuppression in the tumor microenvironment through increased tryptophan metabolism, thereby leading to suppression of antitumoral T-cells (65–68). *IDO1* inhibitors that could restore the anti-tumoral function of T-cells and shift the tumor microenvironment from immunosuppressive to immunogenic are currently under investigation in various clinical trial settings (69, 70). Interleukin 6 (*IL6*), a pleiotropic cytokine, which is an important player in several cellular processes such as proliferation, immune regulation, inflammation, and invasion, has been shown to be upregulated in various cancer types and also predicts poor clinical outcomes in patients with HNSCC (71–74). In our study, we could already show an increase in *IL6* expression in moderate and high-grade dysplastic samples. In accordance with the literature, we also found lymphocyte activation gene-3 (*LAG3*) overexpressed in HNSCC samples. *LAG3* is an immune checkpoint control protein that negatively regulates T-cell and immune response (75). Here, our results demonstrated that *LAG3* upregulation could already be observed in moderate dysplasia. Previously, Choi et al. have demonstrated a stepwise alteration of T-cell repertoires during HNSCC progression, implying changes in the immune microenvironment before malignant invasion (76). In the case of serine peptidase inhibitor Kazal type 5 (*SPINK5)*, its downregulation was even evident in low-grade dysplastic lesions, signifying an early deactivation event in the disease’s evolutionary trajectory. *SPINK5* encodes for a serine protease inhibitor, which plays a crucial role in regulating various cellular processes, including tissue remodeling and inflammation. In HNSCC, *SPINK5* has emerged as a critical player, often serving as a tumor suppressor gene (77). This suggests that *SPINK5*’s reduced expression may occur early in the development of HNSCC, potentially contributing to the initiation and progression of the disease.

Our study has several limitations, primarily stemming from the relatively small sample size. We acknowledge that drawing comprehensive conclusions from a cohort of just seven patients is inherently limited. However, we believe that our study adds valuable insights to the limited body of research that has explored the relationship of the genetic landscape with immune microenvironmental changes in p16-negative HNSCC and matching premalignant and normal squamous mucosa. Additionally, the absence of sequencing data from matched peripheral blood lymphocytes is a constraint, as it hampers the ability to filter out variants originating from clonal hematopoiesis of indeterminate potential (78). Nonetheless, we made substantial efforts to address this issue through rigorous filtering methods, including cross-referencing with the dbSNP database. One notable strength of our study lies in the detailed clinical characterization of the enrolled patients, with extensive follow-up periods and a diverse array of available samples, encompassing, primary and recurrent tumor tissues, as well as different grades of dysplastic lesions and matched normal squamous mucosa. The utilization of archived FFPE tissue aligns with common clinical practice, making our approach more pragmatic for potential clinical implementation.

In summary, findings from this study show that genetic changes and aberrations in immune gene expression become detectable in the early stages of upper aerodigestive tumor development, underscoring the prospect of intervening in the initial disease progression mechanisms. A better understanding of the concept that premalignant lesions may transform presumably with acquired mutations and copy number alterations to escape immune surveillance is crucial. It could provide scientists with new targets for intervention in those patients who are at risk of progression to HNSCC and clinicians with biomarkers for risk stratification and therapy decision-making in patients with high-risk premalignant lesions. In future, a deeper and more comprehensive assessment of epithelial, stromal, and immune heterogeneity in morphologically comparable grades of dysplasia in a cancerized field could potentially help predicting clinical outcomes and guide personalized therapies in patients with high-risk premalignancy. Ultimately, personalized diagnostics to guide treatment decisions, thereby avoiding unnecessary surgical procedures, their associated morbidities as well as reducing health economic costs may only be achieved by larger studies to confirm promising molecular biomarkers.

## Data availability statement

The raw data supporting the conclusions of this article will be made available by the authors, without undue reservation.

## Ethics statement

The studies involving humans were approved by Local ethics committee of the Ludwig-Maximilians-Universität (LMU) in Munich (ref. no. 18-446). The studies were conducted in accordance with the local legislation and institutional requirements. The participants provided their written informed consent to participate in this study.

## Author contributions

AL: Writing – original draft, Writing – review & editing. JK: Conceptualization, Data curation, Formal analysis, Funding acquisition, Investigation, Methodology, Project administration, Resources, Software, Supervision, Validation, Visualization, Writing – original draft, Writing – review & editing. CW: Writing – original draft, Writing – review & editing. AJ: Writing – original draft, Writing – review & editing. PB: Writing – original draft, Writing – review & editing. SF: Conceptualization, Data curation, Formal analysis, Funding acquisition, Investigation, Methodology, Project administration, Resources, Software, Supervision, Validation, Visualization, Writing – original draft, Writing – review & editing.
