# Supplementary figures and images for: Molecular characterization of the evolution of premalignant lesions in the upper aerodigestive tract
Source: Front Oncol. 2024 Apr 19;14:1364958. doi: 10.3389/fonc.2024.1364958 (PMC11067708; doi:10.3389/fonc.2024.1364958)

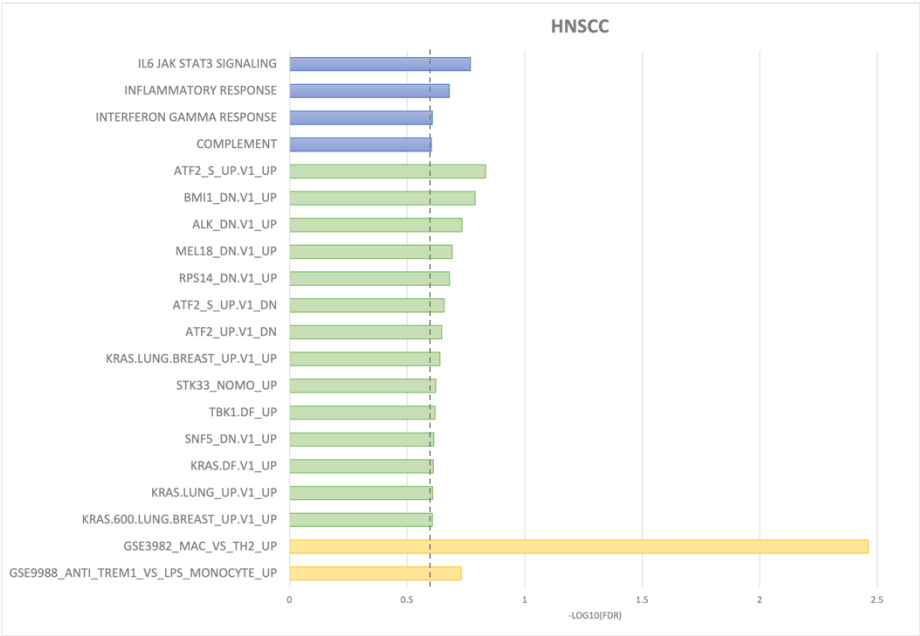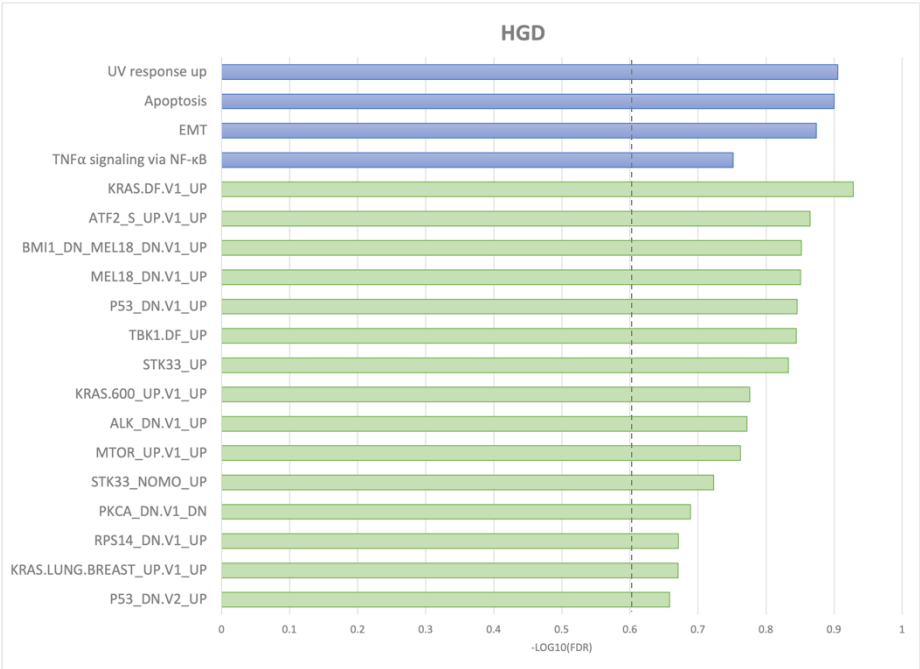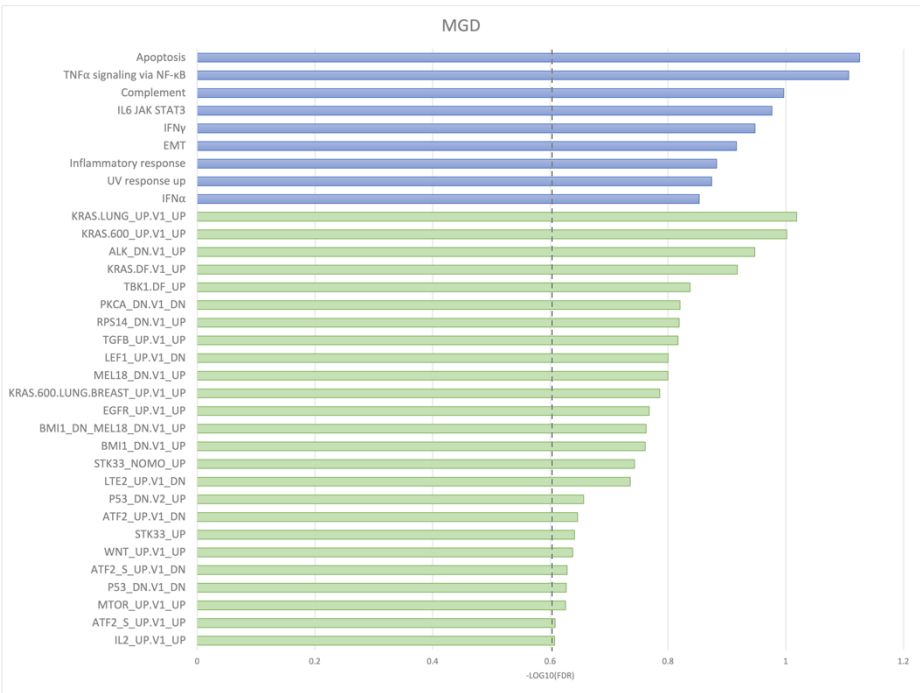

Supplement: Supplementary file 1 [file DataSheet_1.pdf]

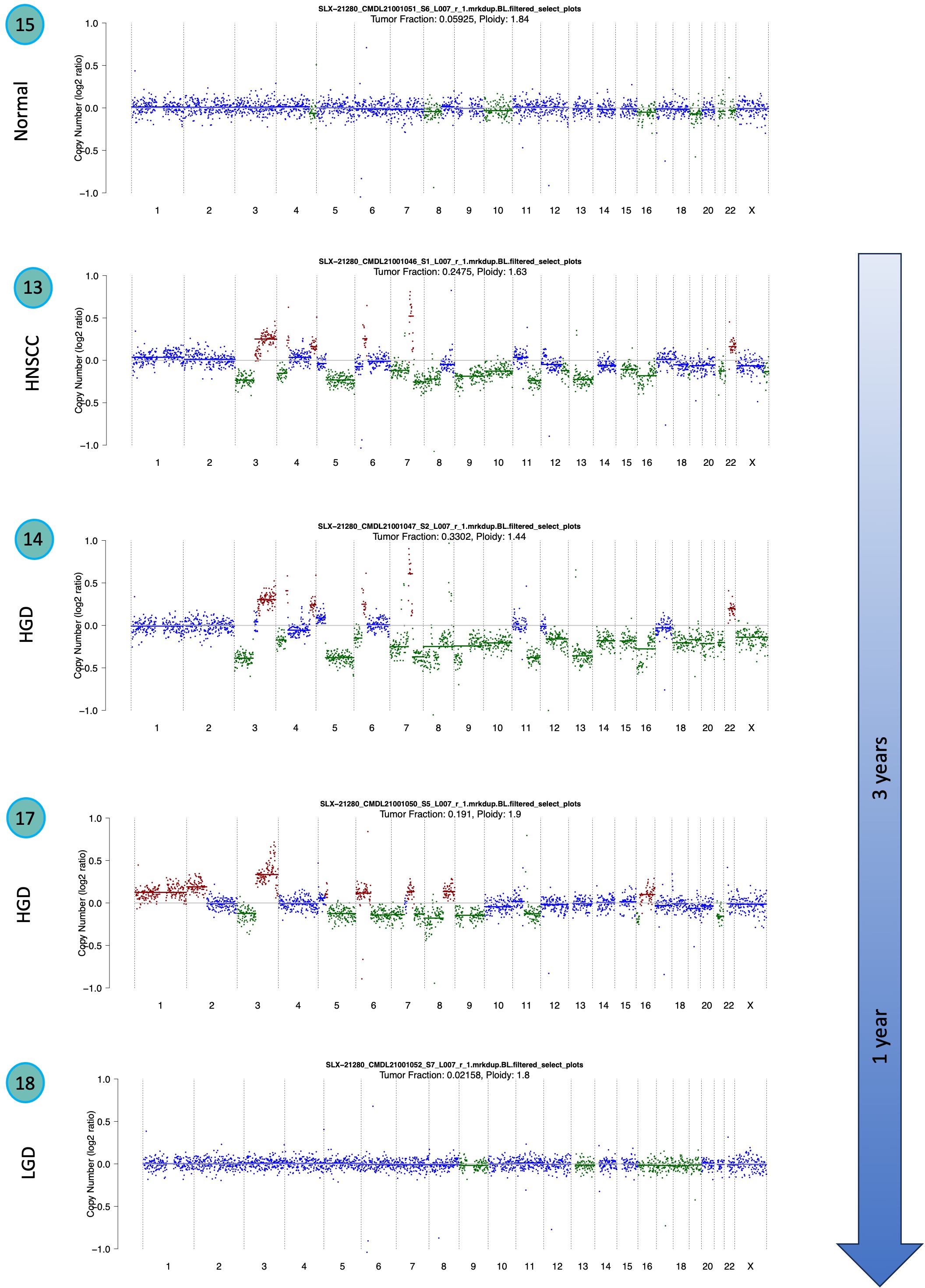

Supplement: Supplementary file 3 [file Image_1.jpeg]

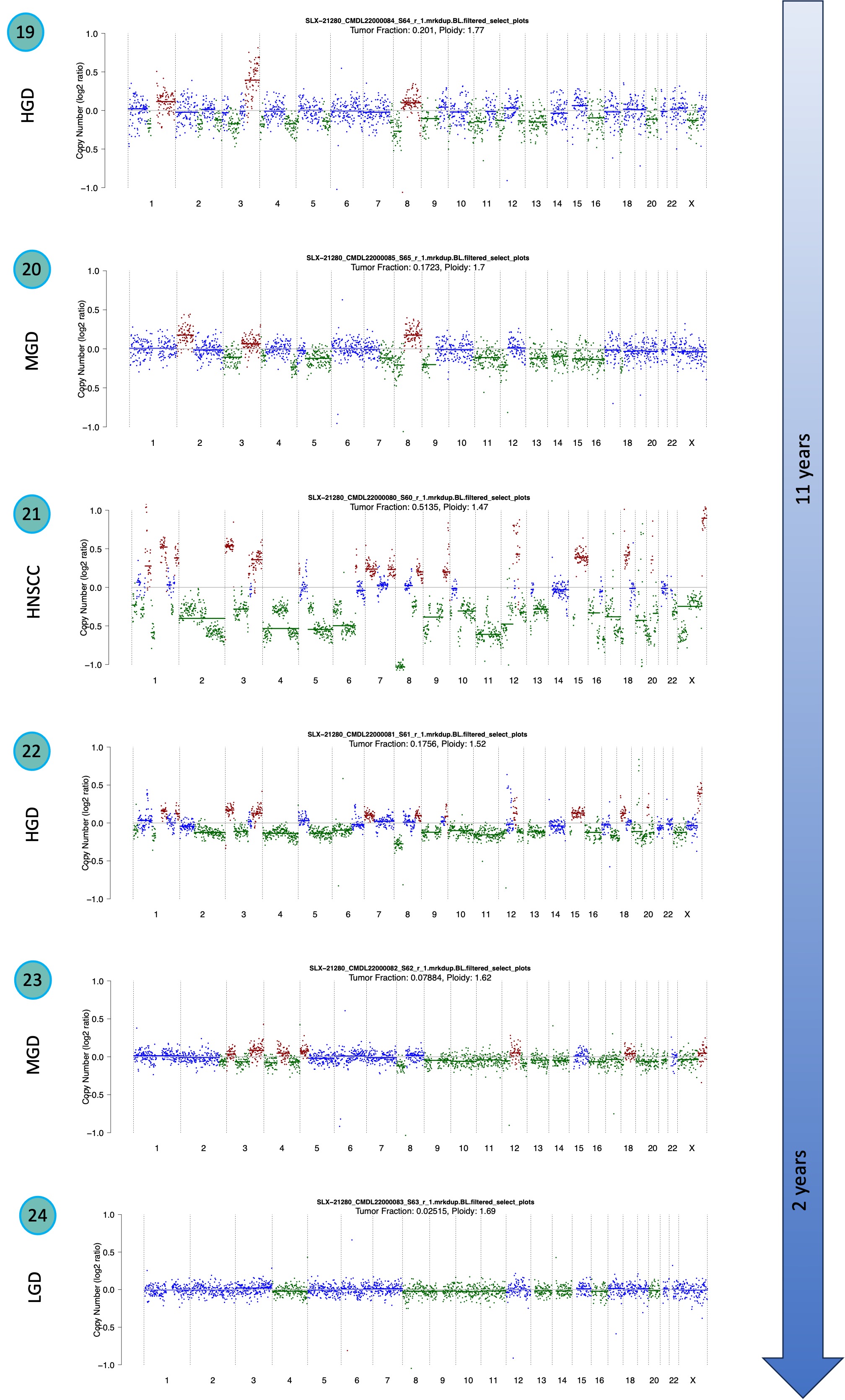

Supplement: Supplementary file 4 [file Image_2.jpeg]

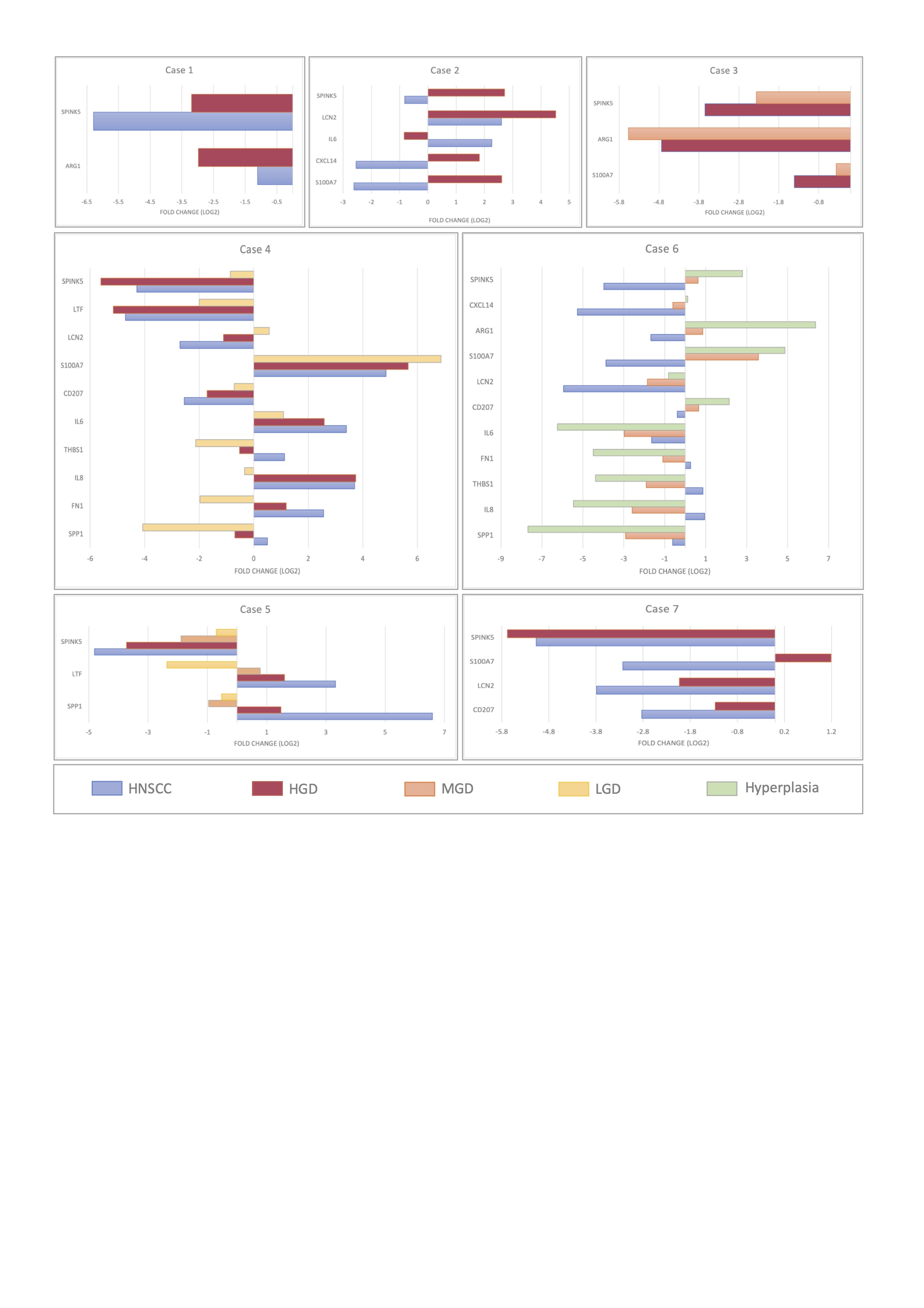

Supplement: Supplementary file 5 [file Image_3.jpeg]

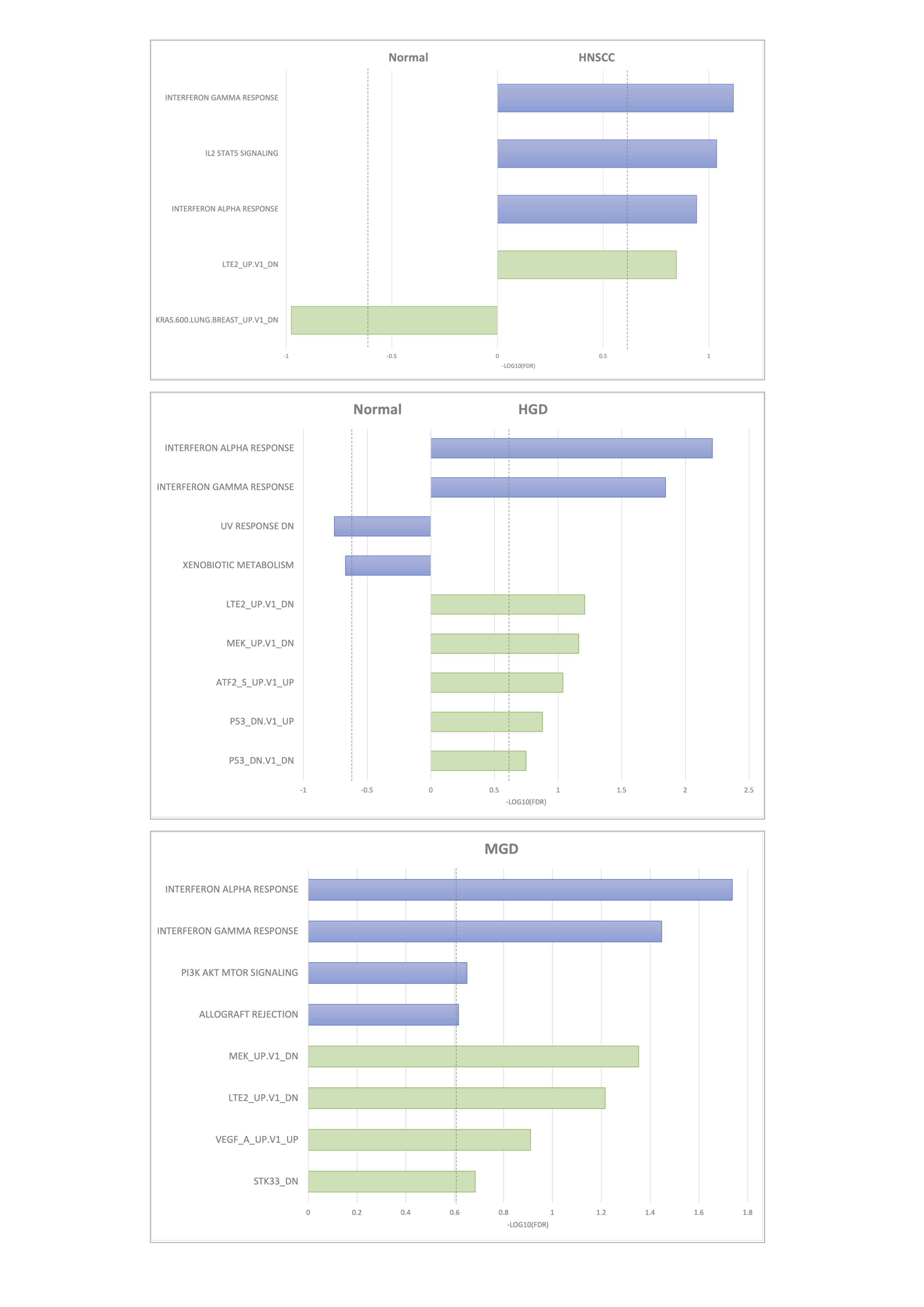

Supplement: Supplementary file 6 [file Image_4.jpeg]
