# Supplementary material for: Molecular characterization of the evolution of premalignant lesions in the upper aerodigestive tract
Source: Front Oncol. 2024 Apr 19;14:1364958. doi: 10.3389/fonc.2024.1364958 (PMC11067708; doi:10.3389/fonc.2024.1364958)
Supplement: Supplementary file 2 [file DataSheet_2.pdf]

HALLMARK_INTERFERON_GAMMA	NES	NOM *p*	FDR
**HNSCC vs. Normal**	1.62	0.03	0.08
**HGD vs. Normal**	1.79	0.007	0.014
**MGD vs. Normal**	1.86	0.004	0.036
**HALLMARK_INTERFERON_ALPHA**	**NES**	**NOM *p***	**FDR**
**HNSCC vs. Normal**	1.68	0.02	0.11
**HGD vs. Normal**	1.91	0.004	0.006
**MGD vs. Normal**	1.85	0.01	0.02
